# Association between Angiotensin-Converting Enzyme-Insertion/Deletion Polymorphism and Diabetes Mellitus-2 in Saudi Population

**DOI:** 10.31557/APJCP.2021.22.1.119

**Published:** 2021-01

**Authors:** Mahmoud Habibullah, Farhana Akter, Xingping Qin, Mohtashim Lohani, Mohammed S Aldughaim, Yahia Al-Kaabi

**Affiliations:** 1 *Department of Medical Laboratory Technology, Faculty of Applied Medical Science, Jazan University, Saudi Arabia. *; 2 *Department of Medicine, University Of Cambridge, United Kingdom. *; 3 *Massachusetts General Hospital Cancer Centre, Harvard Medical School, USA. *; 4 *Emergency Medical Services, Jazan University, Saudi Arabia. *; 5 *King Fahad Medical City, Riyadh, Saudi Arabia. *

**Keywords:** Polymorphism-type 2 diabetes mellitus, angiotensin, converting enzyme gene, Saudi population

## Abstract

**Objectives:**

The association of angiotensin-converting enzyme (ACE) insertion/deletion (I/D) polymorphism and the development of type 2 diabetes mellitus (T2DM) has been debated vigorously but still remains controversial. Therefore, the current study was designed to determine the possible association between ACE I/D polymorphism and T2DM and hypertension (HTN) in a population of Saudi Arabian participants.

**Methods:**

A total of 143 individuals were recruited for the study, consisting of 74 controls and 69 patients with T2DM. Genotyping was performed via polymerase chain reaction.

**Results:**

The genotype frequencies for DD, ID and II in controls were 52.7%, 39.2% and 8.1%, whereas in T2D patients it was 52.2%, 40.6% and 7.2% respectively. The DD frequency was highest out of the three genotypes in both the controls and the T2DM patients.

**Conclusion:**

There was no significant difference found in the genotype and allele frequencies between cases and controls, suggesting that insertion/deletion polymorphism in the ACE gene may not be associated with an increased susceptibility to type 2 diabetes in our study population.

## Introduction

Angiotensin-converting enzyme (ACE) is a zinc-metallopeptidase that plays an essential role in two physiological systems: the production of angiotensin II and breakdown of bradykinin. ACE is also involved in the renin-angiotensin system (RAS) that regulates blood pressure (Sturrock et al., 2013). Studies have suggested that blockage of RAS is associated with a reduced incidence of type 2 diabetes mellitus (T2DM)-associated complications. In a number of these studies, ACE inhibitors were shown to provide renal protection in Bergamo Nephrologic Diabetes Complication Trials (BENEDICT) (Ruggenenti et al., 2004) and ADVANCE (Action in Diabetes and Vascular Disease: Preterax and Diamicron MR Controlled Evaluation) studies (Ruggenenti et al., 2004; Ninomiya et al., 2009). Renin is released by the juxtaglomerular cells in the kidneys following volume loss, salt loss or sympathetic activation, and catalyses the formation of angiotensin I from angiotensinogen. ACE, found mainly in the lungs, converts angiotensin I to angiotensin II, the latter of which is a potent vasoconstrictor, responsible for stimulating the synthesis and release of aldosterone from the adrenal cortex, which increases water and sodium retention, and therefore increases blood volume and blood pressure (Thatcher, 2017). ACE levels are under genetic control and the gene coding for ACE is located on chromosome 17 (region 17q23) with 26 exons and 25 introns (Rigat et al., 1990). A common variant of this gene is known as the insertion/deletion (I/D) variant, with the presence of the ACE D/D genotype being linked to a two-fold increase in ACE activity compared to the I/I genotype (Rigat et al., 1990). Moreover, the frequency of the different ACE alleles are known to vary between different ethnic groups (Barley et al., 1994). Due to the central role of ACE in the RAS, numerous studies have addressed the role of the I/D polymorphism in microvascular disorders. The aim of our study was thus to discover the ACE allele and genotype frequencies among a group of patients with T2DM from Saudi Arabia. 

## Materials and Methods

*Ethical Considerations and Participants*

The study included 143 participants that were attending the department of the King Fahd University Hospital at Al-Khobar between September 2016 and July 2017. The subject group was comprised of newly diagnosed T2DM patients. The participants signed an informed consent and ethical approval form, which was obtained from the institutional clinical research review committee. The patients were diagnosed with T2DM based on the American Diabetes Association (ADA) criteria.

*Inclusion Criteria and Study Groups*

Participants included subjects with newly diagnosed T2DM, defined by the ADA criteria as a fasting blood glucose level > 7.0 mmol/l (American Diabetes Association, 2016). Healthy individuals were randomly selected from volunteers and PSAU staff members without age or sex matching. Their inclusion criteria were defined as them having a fasting blood glucose level <7.0 mmol/l, a negative family history of diabetes, and absence of any medication intake at the time of enrollment.

The study included the following two groups: Control group (n=74) and T2DM group (n=69). A questionnaire in Arabic and English language was completed by each participant to obtain information regarding socio-demographic status, history of diabetes and other co-morbidities. Weight and height data of all participants were determined to compute their body mass index (BMI) using the formula, weight (kg)/height (m)^2^.

*Biochemical Analysis of Blood Samples*

The plasma was removed from the blood samples by centrifugation at 1,000 × g (4°C) for 10 min and maintained at −30°C. Methods of analysis included measuring triglyceride (TG), high-density lipoprotein cholesterol (HDL-C), and total cholesterol (TC) levels. The low density lipoprotein cholesterol (LDL-C) was calculated using Friedewald formula (Tremblay et al., 2004). Lipid profile results were classified according to the Third Report of the National Cholesterol Education Program Guidelines (Adult Treatment Panel III, JAMA. 2001). 

*Genotype Determination*

The genomic DNA was extracted using saliva self-collection kits following the manufacturer’s instructions (DNA Genotek, Inc., Kanata, ON, Canada), assessed for purity and stored at −80°C until further analysis. The ACE gene I/D polymorphism was determined using fragment amplification with the polymerase chain reaction (PCR) technique followed by gel electrophoresis. The flanking primer pairs were as follows: Forward, 5′-GCCCTGCAGGTGTCTGCAGCATGT-3′ and reverse, 5′-GGATGGCTCTCCCCGCCTTGTCTC-3′ (Knoell et al., 2009). Fragment amplification was performed following the protocols explained by Knoell et al., (2009).

**Figure 1:**
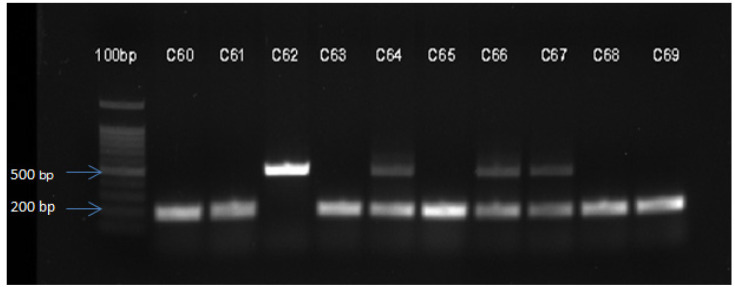
Agarose Gel Electrophoresis Patterns of ACE I/D Gene Polymorphism. Lane number C60, 61, 63, 65, 68 and 69 represent DD homozygotes; lane 62 represents II homozygote and lane 64, 66 and 67 represent I/D heterozygotes

The desired DNA fragments were separated using agarose gel electrophoresis in an electrophoresis tank. The PCR product and loading dye were mixed and loaded to 2% ethidium bromide gel. After 60 min at 120-V current, the gel was removed and visualized using a ChemiDoc™ MP Gel imaging System (Bio-Rad Laboratories, Inc., USA) under UV light. The initial PCR results revealed three genotypes: A 490-bp band (II), a 190-bp band (DD) and the two bands (ID) (ّFigure 1).

*Statistical Analysis*

Statistical analyses were conducted using a Chi square test. The descriptive data and laboratory results were reported as means ± standard deviation. Genotype and allelic frequencies in all groups were reported as numbers and percentages. The genotype data were examined for aberration from that expected using the Hardy-Weinberg equilibrium, and the significant difference between groups was calculated using the *χ*^2^ test of independence. Furthermore, the odds ratios (ORs) and 95% confidence intervals (CIs) were calculated via multiple logistic regression analysis. P<0.05 was considered to indicate a statistically significant difference at 95% CI.

## Results

Average of the baseline characteristics of cases (n=74) were compared with those of controls (n=69) (see Table 1), and, except for the waist circumference and fasting blood glucose level, were not found to be significantly different,.

Average age and standard deviation among the controls was 48.2+9.3 and between cases was 51.6+9.0, but the difference was not found statistically significant (p>0.05). Average height and standard deviation among the controls were 160.9 ± 7.9 and in cases were 161.5 ± 8.3, but the different was not found statistically significant (p>0.05). Average weight and standard deviation among the controls were 80.2 ± 16.4 and in cases were 83.0 ± 17.5, but the different was not found statistically significant (p>0.05). Average BMI and standard deviation among the controls were 30.9 ± 5.9 and in cases were 31.9 ± 6.1, but the different was not found statistically significant (p>0.05).

The average fasting blood glucose level of the controls was 97.1+9.6, whereas for the T2DM group it was 177.4+73.0, which was significantly higher than in the controls (p<0.0001). This was similar to the waist circumference, where the average of the controls was 99.7 ± 13.4, whereas for T2DM group was 105.5 ± 13.1, which was significantly higher than in the controls (p<0.002).

The expected frequencies of ACE gene I/D polymorphism genotype were calculated in accordance to the Hardy-Weinberg equilibrium in both of the studied groups (see Table 2). The genotype frequencies were almost same when compared between both the study groups, whereas the D/D polymorph frequencies were the highest i.e., 39 (52.7%) in the control and 36 (52.2%) in T2DM, followed by the adjusted odds ratio and its 95% confidence interval 1.01 (0.65-1.57) p-value >0.05, and finally I/D was 29 (39.2%) in the control and 28 (40.6%) in T2DM. Adjusted odds ratio and its 95% confidence interval 0.46 1.29 (0.54-3.11) and the I/I genotype frequencies were least in both the groups i.e., 6 (8.1%) in the control and 5 (7.2%) in T2DM) (see Table 3).

**Table 1:** Baseline Characteristic of the Study Population

Variables	Control (n=74)	T2DM (n=69)	P
Gender (Male/Female)	25/49	36/33	-
Age (years)	48.2 ± 9.3	51.6 ± 9.0	0.34
Height (3)	160.9 ± 7.9	161.5 ± 8.3	0.654
Weight (22)	80.2 ± 16.4	83.0 ± 17.5	0.275
Body mass index (kg/m^2^)	30.9 ± 5.9	31.9 ± 6.1	0.327
Waist circumference (3)	99.7 ± 13.4	105.5 ± 13.1	0.002
Fasting glucose (mg/dL)	97.1 ± 9.6	177.4 ± 73.0	0.0001

Data are (mean ± SD) or percentage; T2DM, type-2 diabetes mellitus.

**Table 2:** ACE Insertion/Deletion Polymorphism and Hardy-Weinberg Equilibrium

Genotype	Control (n=74)		T2DM (n=69)		Total(n=143)	
	Observed frequencies	Expected frequencies	Observed frequencies	Expected frequencies	Observed frequencies	Expected frequencies
*D/D*	39(52.70%)	38.7(52.30%)	36(52.17%)	36.2(52.46%)	75(52.45%)	74.9(52.38%)
*I/D*	29(39.19%	29.6(40.00%)	28(40.58%)	27.5(39.86%)	57(39.86%)	57.2(40.00%)
*I/I*	6(8.11%)	5.7(7.70%)	5(7.25%)	5.3(7.68%)	11(7.69%)	10.9(7.62%
*P*-value	>0.05		>0.05		>0.05	

**Table 3:** Allelic and Genotypic Frequencies of the Insertion/Deletion Polymorphism in the *ACE* Gene in Patients with Type-2 Diabetes (T2DM) and Healthy Control Group

	Total n (%)	Control n (%)	T2DM n (%)	*OR (95% CI) *P*	**OR (95% CI) *P*
Genotype					
* D/D*	75 (52.4)	39 (52.7)	36 (52.2)	Reference	Reference
* I/D*	57 (39.9)	29 (39.2)	28 (40.6)	1.04 (0.52-2.08) 0.97	1.01 (0.65-1.57) 0.84
* I/I*	11 (7.7)	6 (8.1)	5 (7.2)	0.90 (0.25-3.21) 1	1.29 (0.54-3.11) 0.46
* I/D + I/I*	68 (47.5)	35 (47.3)	33 (47.8)	1.02 (0.52-1.97) 0.92	1.04(0.68-1.58) 0.85
Allele					
D	207 (72.4)	107 (72.3)	100 (72.5)	Reference	Reference
I	79 (27.6)	41 (27.7)	38 (27.5)	0.99 (0.59-1.67)	1.1 (0.48-2.01)

*Crude odds ratio and (95% CI); ** Age, gender and BMI adjusted odds ratio and (95% CI).

## Discussion

Diabetes mellitus is a major health concern in Saudi Arabia, with a prevalence rate of 24% of the total population (Robert et al., 2017). Of these, 90% have the non-insulin-dependent diabetes mellitus; or T2DM. The etiopathogenesis of T2DM is complex, with both environmental and genetic factors thought to play a role. The underlying genetic factors associated with T2DM are, however, complex and yet to be fully discovered. That said, there is evidence of an increased risk of developing T2DM in patients with the ACE I/D polymorphism (Thomas et al., 2001; Al-Saikhan et al., 2017). There is also evidence of an increase in diabetes induced microvascular complication such as retinopathy (Luo et al., 2016) and nephropathy (Movva et al., 2007). Multiple studies have also reported a direct link between the ACE gene and obesity, and metabolic syndrome, due to increased adipocyte growth and function (Jones et al., 1997; Xi et al., 2012). 

The presence or absence (insertion or deletion) of a 287-bp Alu sequence in the 16th intron of the ACE gene, which present on the 17th chromosome, has been strongly debated in terms of its association with the increased risk of diabetes and diabetic complications (Marre et al., 1994), myocardial infarction (Samani et al., 1996) and HTN (Zee et al., 1992). A number of studies have linked the D/D genotype with such manifestations (Marre et al., 1994; Daimon et al., 2003), while many studies have also denied the associated between the DD genotypes, and development of HTN (Chiang et al., 1997) and diabetes (Daimon et al., 2003)

The link between the ACE I/D polymorphism and the prevalence of T2DM developing in the Saudi Arabian population remains controversial. A recent study showed that the ID/DD genotype and the D allele of the ACE gene I/D polymorphism were strongly associated with the risk of T2DM and HTN developing in the Saudi Arabian population (Al-Saikhan et al., 2017). The contradictory results regarding the involvement of the ACE I/D polymorphism in T2DM are thought to be due to variance in ethnicity and/or gender. 

In order to clarify the role of ACE gene polymorphism in causation of T2DM, we performed the current study in Saudi Arabian T2DM patients with or without HTN. Our findings strongly suggest no association between the DD homozygous genotype and (primarily) the D allele of the ACE gene with T2DM and HTN in Saudi Arabian subjects, when compared with the healthy control group (see Table 3). However, previous studies in other populations have shown a high prevalence of the DD genotype (93.33%) and D allele (81.39%) of the ACE gene in diabetic and HTN patients as compared with control subjects (Jeng et al., 1997; Daimon et al., 2003). In the present study, the frequency of the D/D allele was found to be almost the same in T2DM patients with or without HTN compared with the control subjects (P<0.0001).

Unfortunately, we were not able to assess the role of other genetic polymorphisms on T2DM in this population. Furthermore, we only assessed a subgroup of the Saudi population, and therefore our results cannot be generalized to the entire population. We believe further studies investigating different ethnic groups will provide more valuable information than investigating patients from the same ethnic group, even within the same country, and thus provide clues to potential specific biomarkers. 

In conclusion, the association between ACE gene I/D polymorphism and type 2 diabetes is not conclusive, as the inconsistent observations between ACE gene polymorphism and T2DM may be due to ethnic and geographical variations. In this study we investigated the possible relationship between ACE gene I/D polymorphism and diabetes in a group of patients from Saudi Arabia. PCR (polymerase chain reaction) was used to detect the ACE gene I/D polymorphism in T2DM patients and metabolic measurements, including blood glucose. Frequencies of the ACE genotypes (DD, ID, and II) were not significantly different between patients with T2DM and the non-T2DM control. In conclusion, the relationship of ACE gene I/D polymorphism with T2DM is insignificant in Saudi patients with T2DM. 
